# Risk stratification and source classification in *Staphylococcus aureus* bloodstream infections: high mortality linked to mrsa, biomarkers, and treatment efficacy

**DOI:** 10.3389/fimmu.2026.1790382

**Published:** 2026-06-05

**Authors:** Qiangsheng Feng, Xiaoqin Ha, Yuejuan Song

**Affiliations:** Department of Clinical Laboratory, The 940th Hospital of Joint Logistics Support Force of People’s Liberation Army, Lanzhou, China

**Keywords:** BSIs, clinical characteristics, mortality, risk factors, *S. aureus*, survival analysis

## Abstract

*Staphylococcus aureus* bloodstream infection (SAB) carries a high mortality rate. This study aims to develop a source-specific classification framework to guide management and improve outcomes.

**Methods:**

We conducted a retrospective study of 321 *S. aureus* bloodstream infection patients (2012-2025), using ROC analysis, survival curves, and SPSS to evaluate 12-month in-hospital mortality and its predictors.

**Results:**

Among 3,516 bloodstream infections, 321 episodes of *S. aureus* bacteremia (SAB) were identified (9.12%). The median time to positivity was 14 hours. The most common infection sources were the lungs (26.8%), catheter-related infections (24.3%), and skin/soft tissue infections (23.4%). Hemodialysis was the predominant comorbidity (21.2%). Biomarker analysis showed high diagnostic accuracy for PCT (AUC 0.957), WBC (AUC 1.000), and neutrophil percentage (AUC 0.913). MRSA accounted for 38% of isolates. Both MRSA and MSSA exhibited >50% resistance to penicillin, erythromycin, and clindamycin, but none to linezolid, vancomycin, or tigecycline. Overall, in-hospital mortality was 30.2%, significantly higher in MRSA (38%) than in MSSA (26%) (p=0.048). Notably, 36.1% of deaths occurred within 24 hours, and 58.7% within 7 days. The 10-day survival rate was lower in MRSA (0.609 vs. 0.759, p=0.029). Healthcare-associated infections (58%) showed no significant association with MRSA prevalence after Bonferroni correction. Independent mortality predictors included qSOFA score ≥2 (OR 3.450, 95% CI 1.835-6.835, p<0.001), age ≥60 years (OR 2.654, 95% CI 1.628-4.327, p<0.001), pulmonary source of BSI (OR 2.362, 95% CI 1.407-3.963, p<0.001), neutrophil percentage ≥89.60% (OR 2.195, 95% CI 1.294-3.720, p=0.003), procalcitonin ≥1.84 ng/mL (OR 2.152, 95% CI 1.210-3.824, p=0.008), ineffective or no antibiotic use (OR 2.359, 95% CI 1.176-4.730, p=0.008), and MRSA infection (OR 1.756, 95% CI 1.081-2.853, p=0.023).

**Conclusion:**

This study establishes high-accuracy biomarkers (PCT/WBC) and key mortality predictors for *S. aureus* BSIs, including MRSA infection, qSOFA≥2, and ineffective antibiotics. These findings enable early risk stratification and emphasize the source-specific classification for prompt, appropriate antimicrobial therapy to improve clinical outcomes.

## 1 Introduction

*Staphylococcus aureus* bloodstream infection (SAB), confirmed by positive blood culture, represents a leading cause of global bacteremia-associated mortality, with an estimated case fatality rate of 15–30% accounting for approximately 300,000 deaths annually (1). Biomarkers including procalcitonin (PCT), C-reactive protein (CRP), and interleukin-6 (IL-6) serve as dynamic indicators for monitoring disease progression and therapeutic response in SAB (2, 3). Clinical management of confirmed SAB requires systematic risk stratification, encompassing factors such as indwelling devices (e.g., cardiac implants and central venous catheters), prosthetic material implantation, recent invasive procedures or trauma, hemodialysis dependence, diabetes mellitus, and history of *S. aureus* infection (4, 5). Furthermore, *S. aureus* is a predominant pathogen in community-acquired pneumonia and skin and soft tissue infections (6, 7).

Although *S. aureus* commonly colonizes humans as a commensal, it is also a frequent pathogen capable of causing invasive infections across multiple organ systems, including the skin, soft tissues, bones, bloodstream, and respiratory tract (8). Bacteremia typically arises from uncontrolled localized infection, underscoring the critical importance of source control. Evidence indicates that delayed source control—for instance, a median delay of 3 days compared to 1 day—is associated with prolonged bacteremia and elevated mortality, with each additional day increasing the relative risk of death (9). Similarly, in catheter-related SAB, delayed catheter removal beyond 3 days is linked to higher relapse rates (10). The clinical severity of SAB is further exemplified by specific manifestations such as bacteremic pneumonia, which carries a 30-day mortality rate as high as 46.9% (11). Patient-specific factors also significantly impact outcomes; methicillin-resistant *S. aureus* (MRSA) infections, for example, are more frequently identified in older patients with greater comorbidity burdens (12).

Given the heterogeneous nature of *S. aureus* infections, management strategies should be individualized. For instance, routine local antibiotic administration in paediatric acute haematogenous osteomyelitis is discouraged due to unproven efficacy and significant potential harms, including surgical risks, toxicity, and added cost (13). In this context, the present study seeks to develop a refined classification framework for *S. aureus* bloodstream infections—such as those originating from pulmonary sites—and to establish source-specific therapeutic guidance to improve patient outcomes.

## 2 Materials and methods

### 2.1 Study design

### 2.2 Study population

Patients with bloodstream infections (BSIs) diagnosed between January 1, 2013 and December 31, 2024 were included, all patients were followed for 12 months after the date of first positive blood culture (or until death). Survival status was ascertained through hospital electronic records and outpatient clinical notes. This study conducted a retrospective review of clinical data from patients diagnosed with *S. aureus*-BSIs patients at the 940th Hospital of the Joint Logistics Support Force of the People’s Liberation Army. Statistical analyses were performed on factors such as antimicrobial therapy (Vancomycin VS Levofloxacin or moxifloxacin VS Cephalosp or carbapenem), infection site, underlying diseases, and in-hospital mortality.

### 2.3 Inclusion/exclusion criteria

Selection criteria for the inclusion of patients with *S. aureus*-BSIs. A total of 354 cases of *S. aureus*-BSIs with positive blood culture evidence were, thirty-three (cases) were excluded due to a repeated history of blood culture. Patients with hematological diseases and agranulocytosis were not included in the calculation of WBC and NEU%.

### 2.4 Definitions

Infection Source definitive confirmation requires isolation of the same pathogen species from both blood and a suspected focal site, with a fully compatible antimicrobial resistance profile — i.e., identical susceptibility results for key antibiotic classes (carbapenems, third−generation cephalosporins, fluoroquinolones, and aminoglycosides). Minor discrepancies in other antibiotics are acceptable If microbiological confirmation is absent, a source is designated as probable based on a clear invasive imaging finding (e.g., CT or ultrasound evidence of an abscess) from a site consistent with the patient’s clinical signs, symptoms, and risk factors. According to the CLSI M100 definition, MRSA refers to *S.aureus* isolates that are resistant to oxacillin. The resistance mechanism typically involves acquisition of the mecA or mecC gene, which encodes PBP2a (penicillin−binding protein 2a). This protein has reduced affinity for all β−lactam antibiotics, including penicillins, cephalosporins, and carbapenems, rendering them clinically ineffective. Consequently, CLSI considers MRSA to exhibit a class−effect resistance to all β−lactam agents (including cephalosporins and carbapenems). IDSA Definition of Complicated vs. Uncomplicated *S.aureus* Bacteremia (SAB) (14). According to the 2011 IDSA MRSA guidelines, uncomplicated SAB requires all: endocarditis excluded, no prostheses, negative follow-up blood cultures, fever resolution within 72 hours, and no metastatic infection. Complicated SAB is any case failing these criteria, including endocarditis, metastatic infection, implanted prostheses, positive follow-up cultures after 48 hours, or systemic infection signs. Risk factors (community acquisition, persistent bacteremia, prosthetic material, systemic skin findings) also indicate complicated SAB, guiding treatment duration (4–6 weeks for complicated vs. 14 days for uncomplicated). Ineffective antibiotic therapy was defined as administration of an antimicrobial agent to which the isolated pathogen was resistant according to *in vitro* susceptibility testing (CLSI breakpoints).No antibiotic therapy was defined as absence of any antibiotic treatment within 48 hours after the first positive blood culture.

### 2.5 Variables

Demographic and clinical characteristics were analyzed as exposure variables. This included age, sex, Use of antimicrobial agents, antimicrobial therapy therapy (sensitive antibacterial drugs: Vancomycin VS Levofloxacin or moxifloxacin VS Cephalosp or carbapenem), ICU admission, Administration time (sensitive antibacterial drugs), MRSA, Pathogen source, and Hematologic disease or malignant tumor.

### 2.6 Data collection

We retrieved clinical and laboratory data of patients with *S. aureus*-BSIs from the hospital’s administrative system, the Laboratory Information System (LIS), and the digital medical record system. This comprehensive data collection ensured complete etiological documentation, even for patients who were discharged during the study period.

### 2.7 Endpoint

The 28−day mortality endpoint was used as the primary outcome for logistic regression; the 12−month follow−up was used for Kaplan−Meier survival analysis.

### 2.8 Ethical oversight

The study was approved by the Ethics Committee of the 940th Hospital of the Joint Logistics Support Force of the People’s Liberation Army. The committee waived the need for informed consent. The study adhered to the ethical standards outlined in the Declaration of Helsinki (1975) and its amendments.

### 2.9 *S. aureus*-BSIs culture, identification and antimicrobial susceptibility test

All the subjects recorded episodes who were hospitalized and suspected of BSIs between January 2018 and January 2025, blood cultures were obtained using BacT/ALERTblood culture bottles (bio-Mérieux, Inc, Durham, NC) or BD FA and SN blood culture bottles and incubated in the BacT/ALERT 3D (bioMérieux, Inc) or BD FX 400 automatic monitoring system for a week in the clinical microbiology laboratory of the hospital. When Bottles flagged as positive after Gram positive cocci (quadruple), report the critical value and switch to blood culture onto blood agar and eosin−methylene blue (EMB) agar and incubated at 35 °C CO_2_ for 24h. After colony formation, microbial identification was performed using the corresponding GP card on the VITEK Compact-II automatic microorganism identification system or matrix−assisted laser desorption/ionization time−of−flight mass spectrometry (MALDI−TOF MS) (Yixin Bochuang, China) according to the manufacturer’s instructions, ascore of ≥25 was accepted for species−level identification. Antimicrobial susceptibility test was used AST−GP67 card in VITEK 2 system (bioMérieux, Inc, Durham, NC) Interpretive criteria for antimicrobial susceptibility testing refer to CLSI M100.

### 2.10 Statistical analyses

Statistical analyses were conducted using SPSS 22.0, with statistical significance set at p < 0.05. Factors associated with in-hospital mortality were illustrated using a forest plot (p < 0.01). Kaplan-Meier survival curves were used to estimate survival rates, and the sensitivity and specificity of clinical biomarkers were assessed using receiver operating characteristic (ROC) curves.

## 3 Results

### 3.1 Clinical characteristics and diagnostic biomarker performance in patients with *S. aureus*-BSI

Of 3,516 confirmed bloodstream infections patients, 728 positive blood cultures yielded *S. aureus*, representing 321 distinct episodes of SAB. Data were obtained from 728 positive blood culture bottles (392 aerobic, 324 anaerobic, and 12 pediatric) collected between 2013 and 2025. The median time to culture positivity was 14 hours (IQR: 10-23). Patients had a mean age of 51.5 ± 21.5 years (range: 3-85) with a male-to-female ratio of 2.7:1. *S. aureus* BSI accounted for 9.12% (321/3,516) of all BSI cases.

**Figure 1:**
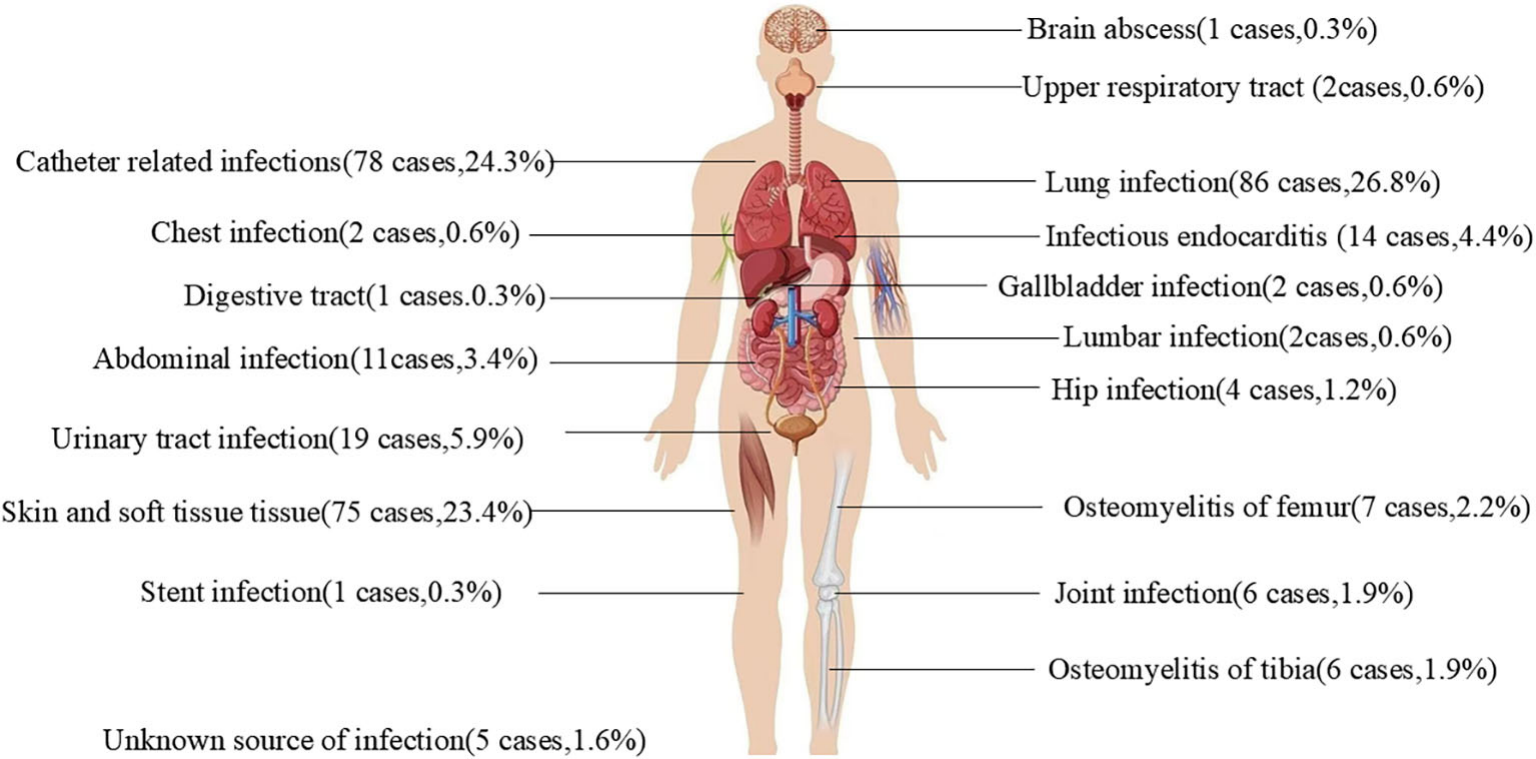
Infection sources in 321 patients with Staphylococcus aureus bloodstream infections (BSIs). The most common sources were lung (26.8%), catheter-related (24.3%), and skin/soft tissue infections (23.4%). A detailed breakdown of all primary and secondary infection sites is provided.

Overall, the most common infection sources were lung infections (26.8%), catheter-related infections (24.3%), skin and soft tissue infections (23.4%), and urinary tract infections (5.9%) (**Figure 1**). Major comorbidities included hemodialysis due to uremia (21.2%), trauma or burns (8.4%), cerebrovascular disease (7.5%), hematological diseases (7.5%), and cancer or malignant tumors (7.2%) (**Table 1**).

**Table 1:** Host factor in S.aureus-BSIs (n=321 cases).

Host factors	cases	%
Hemodialysis patient with uremia	68	21.2
Trauma or burn	27	8.4
Cerebrovascular disease patients	24	7.5
Hematological disease	24	7.5
Cancer or malignant tumor	23	7.2
Osteomyelitis	18	5.6
Type 2 diabetes	18	5.6
Infective Endocarditis	14	4.4
Pulmonary infection	14	4.4
Lumbar spine infection and Joint infection	14	4.4
Decompensatory cirrhosis	12	3.7
Skin and soft tissue infections	9	2.8
Autoimmune Disease	9	2.8
Aortic dissection	5	1.6
Prostatitis	5	1.6
Bile duct stones	4	1.2
Other disease	33	10.3

**Figure 2:**
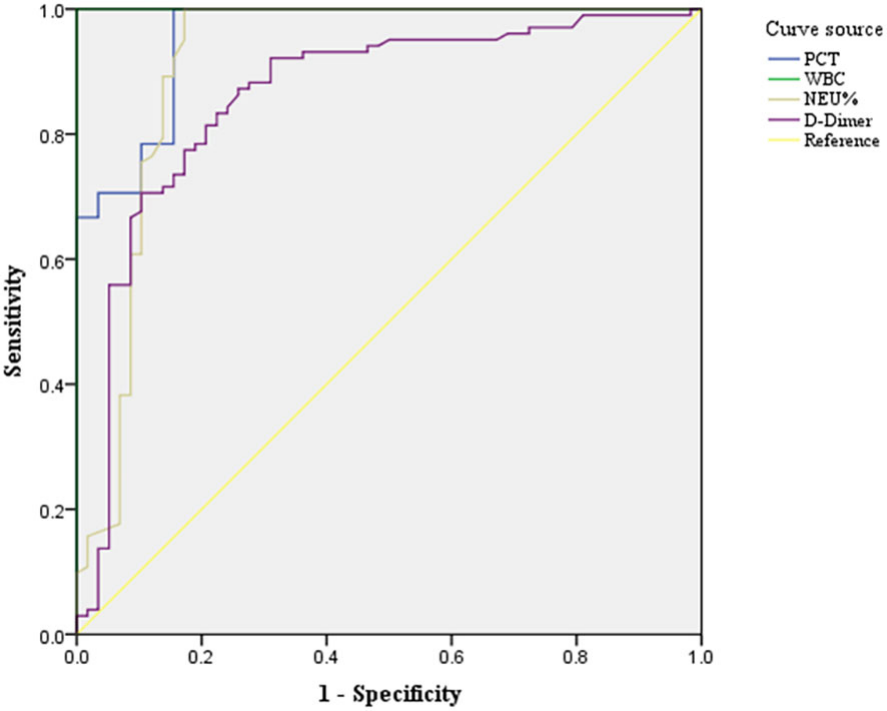
Diagnostic performance of biomarkers for Staphylococcus aureus bloodstream infection. Procalcitonin (PCT), white blood cell count (WBC), and neutrophil percentage (NEU%) showed high accuracy (AUC-ROC: 0.913-1.000). In contrast, qSOFA ≥2 demonstrated low sensitivity (23.8%). Optimal cutoffs and corresponding sensitivity/specificity are detailed.

The discriminatory ability of biomarkers for *S. aureus* -BSIs was assessed in a cohort of 321 patients. Diagnostic performance, evaluated using the area under the receiver operating characteristic curve (AUC-ROC) for each biomarker, is summarized in **Figure 2** and **Table 2**. PCT), WBC), and neutrophil percentage (NEU%) demonstrated high diagnostic accuracy, with AUC-ROC values of 0.957, 1.000, and 0.913, respectively. At the optimal cutoff value of 1.84 ng/mL, PCT exhibited a sensitivity of 1.000 and a specificity of 0.742. WBC achieved perfect discrimination with an AUC-ROC of 1.000; at a cutoff of 11.67 × 10^9^/L, it yielded both sensitivity and specificity of 1.000. NEU% also showed strong performance (AUC-ROC = 0.913), with a sensitivity of 1.000 and a specificity of 0.828 at the cutoff value of 89.60%. In contrast, qSOFA ≥2 showed low sensitivity (23.8%) for predicting *S. aureus* BSI.

**Table 2:** AUC for the biomarker diagnostic value in S.aureus-BSIs (n=321 cases).

Biomarkers	AUC value	Cut-off value	Sensitivity	Specificity	95% Confidence interval	
					Lower	Upper
PCT (ng/ml)	0.957	1.84	1.000	0.741	0.928	0.986
WBC (×10^9^/L)	1.000	11.67	1.000	1.000	1.000	1.000
NEU% (%)	0.913	89.60	1.000	0.828	0.953	0.973
D-Dimer (mg/L)	0.861	1.57	0.804	0.793	0.796	0.926
qSOFA score		≥2	23.8			

PCT, procalcitonin; WBC, White blood cell; NEU%, Neutrophil %. Biomarkers significance differences analysis S.aureus (321cases) VS Control (Non-infected patients,300cases) using Mann-Whitney U test, the *p*-value (<0.001) for PCT [95CI, Z = 9.639, *p* < 0.001, Median 2.38(0.52,12.05)VS 0.10 (0.05,0.32)],25% S.aureus-BSIs patients PCT<0.52 ng/ml;WBC [95CI, Z = 8.57, *p* < 0.001, 11.3(6.90,15.98)VS 7.83 (5.95,9.38)],NEU% [95CI, Z = 16.08, *p* < 0.001, 88.0(83.5,92.5)VS 67.7 (57.6,76.1)] and D-Dimer [95CI,*p* < 0.001, Z = 7.664, *p* < 0.001, 3.84(1.85,6.97)VS 0.47(0.18,1.30)].

### 3.2 Drug resistance rates and treatment outcomes

**Figure 3:**
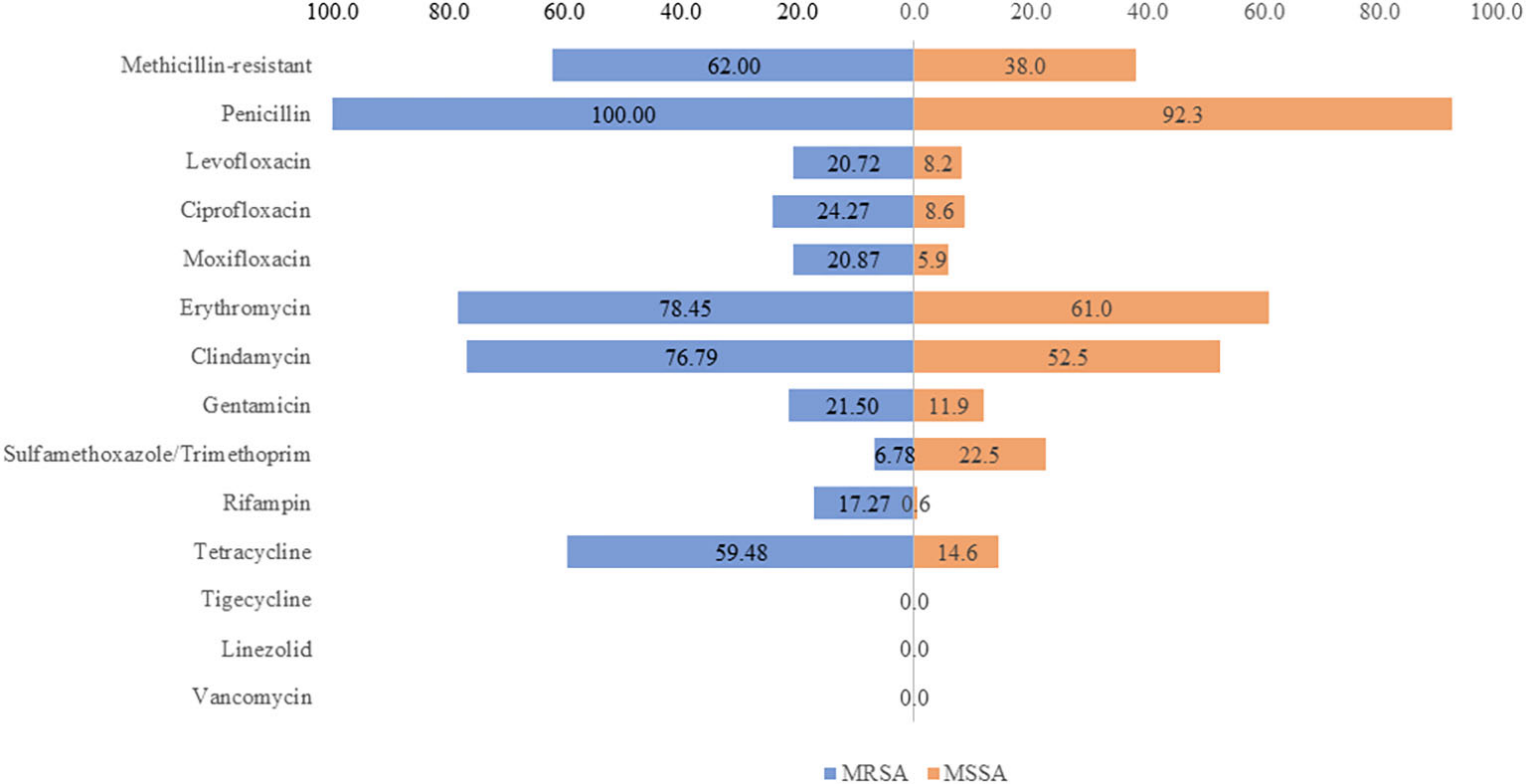
Antimicrobial resistance profiles of 321 S. aureus bloodstream isolates. MRSA accounted for 38% of isolates. Both MRSA and MSSA showed high resistance (≥ 50%) to penicillin, erythromycin and clindamycin, while maintaining susceptibility to linezolid, vancomycin and tigecycline.

Analysis of 321 *S. aureus* isolates from BSIs revealed that MRSA and MSSA accounted for 38% and 62% of the isolates, respectively. Both MRSA and MSSA exhibited high resistance (≥50%) to penicillin, erythromycin, and clindamycin. In contrast, resistance to levofloxacin, ciprofloxacin, moxifloxacin, trimethoprim, rifampicin, and gentamicin was below 30%. No resistance was observed to linezolid, vancomycin, or tigecycline. We compared the resistance rates of MRSA and MSSA to 10 antimicrobial agents using the χ² test with Bonferroni correction (α = 0.05/10 = 0.005). The differences were statistically significant. (**Figure 3**).

**Figure 4:**
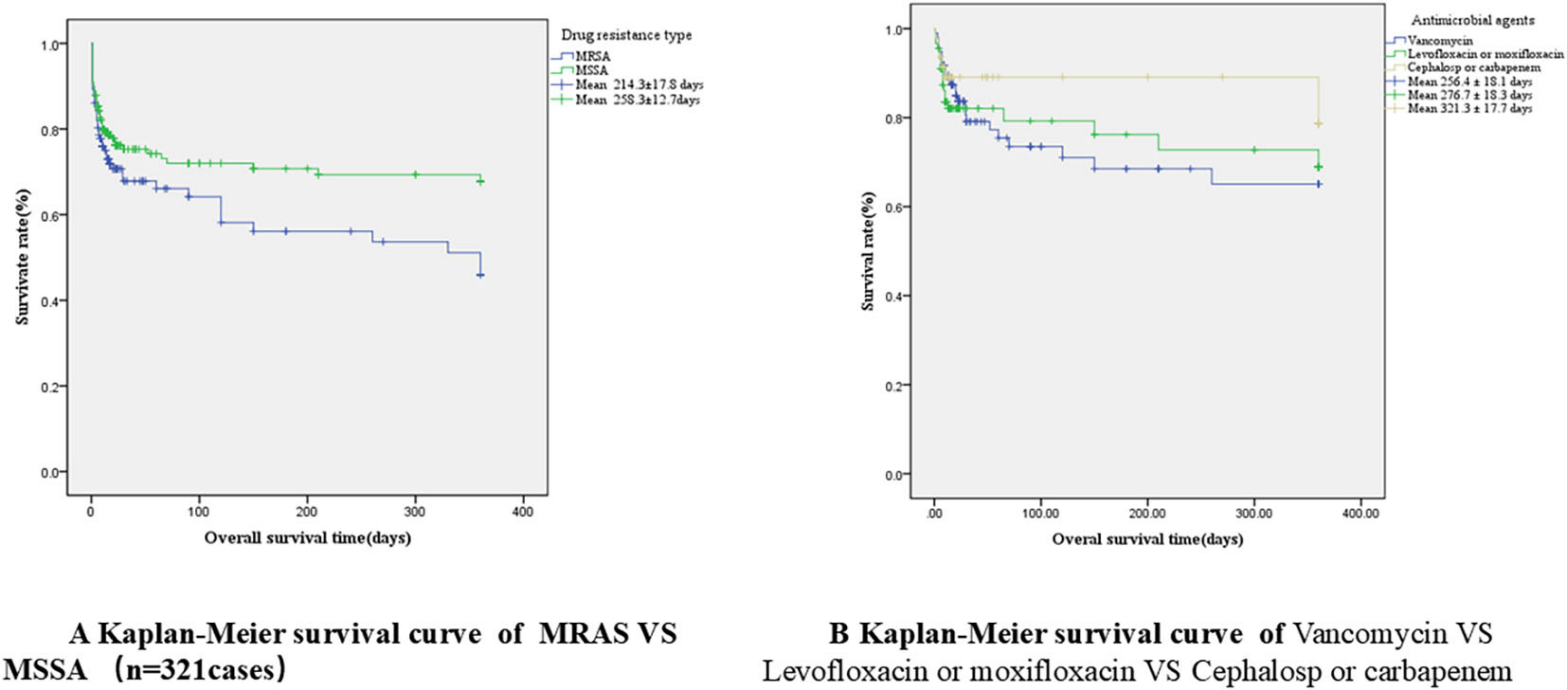
**(A)** Kaplan-Meier survival curve of MRAS VS MSSA (n=321cases). MRSA VS MSSA 10-days survival rate 0.759 ± 0.039 VS 0.798 ± 0.029, 28-days survival rate 0.678 ± 0.046 VS 0.753 ± 0.033.90-days survival rate 0.642 ± 0.050 VS 0.720 ± 0.036. *χ* = 4.758, p=0.029. **(B)** Kaplan-Meier survival curve of Vancomycin VS Levofloxacin or moxifloxacin VS Cephalosp or carbapenem 10-days survival rate 0.918 ± 0.028 VS 0.910 ± 0.030 VS 0.914 ± 0.041, 28-days survival rate 0.806 ± 0.043 VS 0.821 ± 0.0420VS 0.891 ± 0.046, 90-days survival rate 0.735 ± 0.052 VS 0.792 ± 0.049 VS 0.891 ± 0.046, *χ* = 1.802, p=0.406.

Among 321 patients, the overall in-hospital mortality rate was 30.2% (97/321). Mortality was significantly higher in those with MRSA bloodstream infections (BSIs) (38%) compared to those with MSSA BSIs (26%) (χ² = 3.309, p = 0.048). Notably, 36.1% (26/97) of all *S. aureus* BSI-related deaths occurred within 24 hours of infection. An additional 22 deaths (22.7%) occurred between days 2 and 7, and 21 deaths (21.6%) between days 8 and 28. Survival analysis revealed a substantially lower 10-day survival rate in the MRSA group (0.609 ± 0.054) compared to the MSSA group (0.759 ± 0.039; log-rank χ² = 4.758, p = 0.029). Further analysis of 10-day survival based on antibiotic regimens showed no significant differences among treatment groups: vancomycin (0.918 ± 0.028), levofloxacin or moxifloxacin (0.910 ± 0.030), and cephalosporins or carbapenems (0.914 ± 0.041) (log-rank χ² = 1.802, p = 0.406) (**Figures 4A, B**).

### 3.3 Epidemiology of healthcare-associated infections and independent risk factors for mortality in *S. aureus* BSI

**Figure 5:**
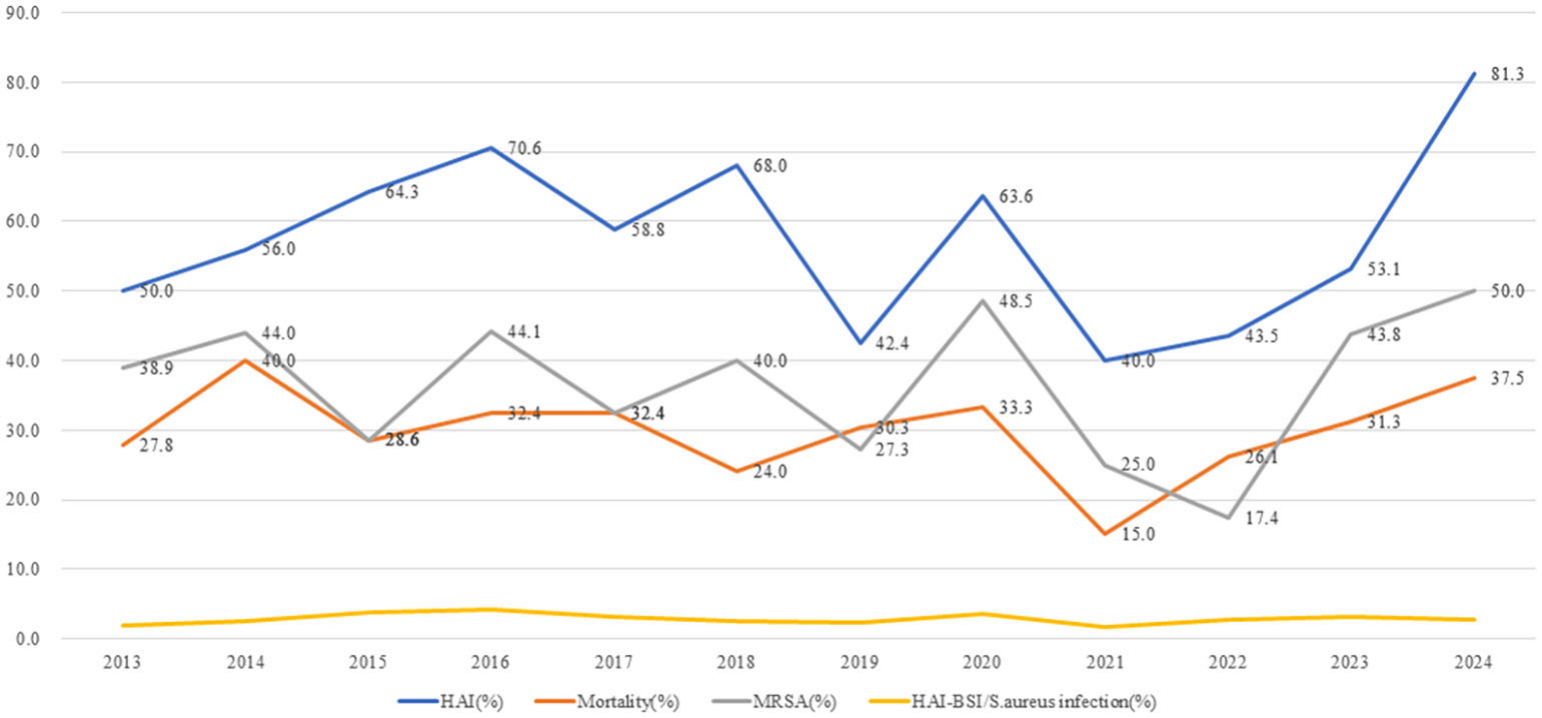
Correlation Among Healthcare-Associated Infections, Mortality, and MRSA Prevalence. Between healthcare-associated infections and MRSA prevalence (R = 0.713, p=0.009), and between MRSA prevalence and mortality (R = 0.628, p=0.029).

**Figure 6:**
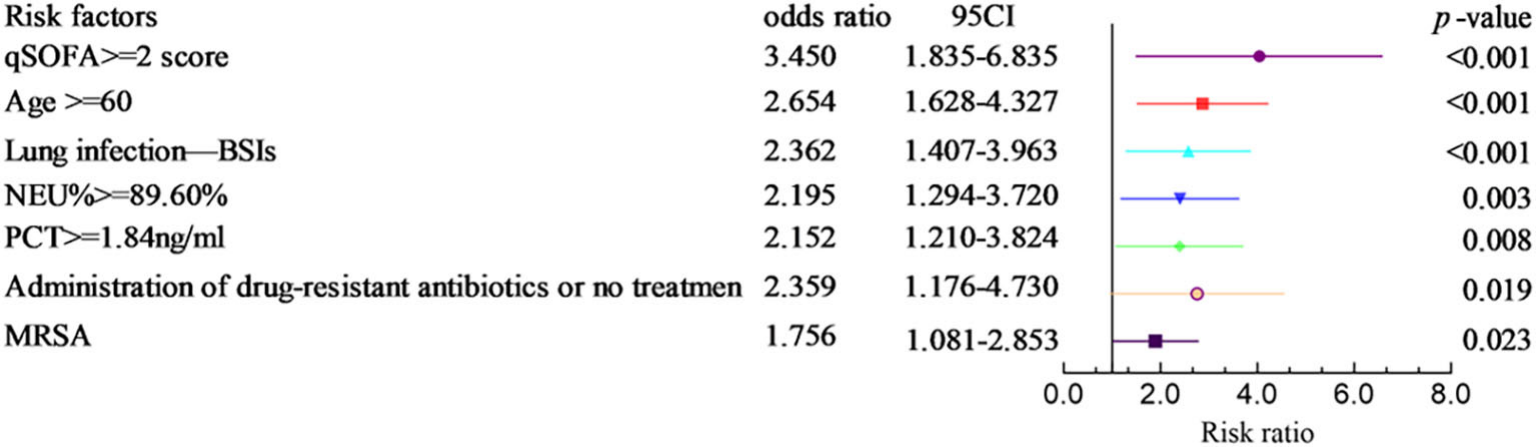
Independent predictors of in-hospital mortality in S. aureus bloodstream infection. Multivariate analysis identified qSOFA ≥2, age ≥60 years, pulmonary source, elevated neutrophil percentage (≥89.60%), high procalcitonin (≥1.84 ng/mL), ineffective/no antibiotic therapy, and MRSA infection as significant risk factors, with odds ratios and 95% confidence intervals shown.

Of the 321 patients, 186 (58.0%) had healthcare-associated infections (HAIs) and HAI-BSI/*S. aureus* infection (2.9%). Among these HAIs, MRSA and MSSA accounted for 45.7% and 54.3% of cases, respectively. The primary sites of HAIs were catheter-related (78 cases), pulmonary (56 cases), skin and soft tissue (24 cases), abdominal (9 cases), and urinary system infections (7 cases). The patient mortality rate showed no significant trend over the last 12 years, χ² test with Bonferroni correction (α = 0.05/12 = 0.004). Although unadjusted correlation analysis suggested positive associations between healthcare−associated infections and MRSA prevalence (2013−2024) and between MRSA prevalence and mortality, these associations were no longer statistically significant after applying the χ² test with Bonferroni correction (α = 0.05/12 = 0.004). (**Figure 5**). Univariate analysis identified multiple factors associated with in-hospital mortality. Subsequent multivariate regression analysis confirmed the following independent predictors of mortality: qSOFA score ≥2 (OR 3.450, 95% CI 1.835-6.835, p<0.001), age ≥60 years (OR 2.654, 95% CI 1.628-4.327, p<0.001), pulmonary source of BSI (OR 2.362, 95% CI 1.407-3.963, p<0.001), neutrophil percentage ≥89.60% (OR 2.195, 95% CI 1.294-3.720, p=0.003), procalcitonin ≥1.84 ng/mL (OR 2.152, 95% CI 1.210-3.824, p=0.008), administration of ineffective or no antibiotics (OR 2.359, 95% CI 1.176-4.730, p=0.008), and MRSA infection (OR 1.756, 95% CI 1.081-2.853, p=0.023). (**Figure 6**; **Table 3**).

**Table 3:** Demographic, clinical and laboratory, findings of patients on admission.

Demographics and clinical characteristics	Total (n=321 cases)	Nonsurvivor	Survivor	*P*
		(n=97(cases)	(n=224cases)	
Sex				*p* = 0.275
Female	86(27%)	30(35%)	56(65%)	
male	235(73%)	67(29%)	168(71%)	
Age				*p<*0.001
>=60	129(40%)	55(43%)	74(57%)	
<60	192(60%)	42(22%)	150(78%)	
Healthcare Associated Infection				*p=*0.349
Yes	186(58%)	60(32%)	126(68%)	
No	135(42%)	37(27%)	98(73%)	
ICU admission				*p* = 0.337
Yes	20(6%)	8(40%)	12(60%)	
No	301(94%)	89(30%)	44(70%)	
Use of antimicrobial agents				*p=*0.019
Sensitivity of antimicrobial susceptibility test	253(86%)	54(21%)	199(79%)	
Administration of drug-resistant antibiotics or no treatmen	41(14%)	16(39%)	25(61%)	
PCT cut-off value				*p=*0.008
>=1.84 ng/ml	131(55%)	51(39%)	80(61%)	
<1.84 ng/ml	105(45%)	24(23%)	81(77%)	
WBC cut-off value				*p=*0.069
>=11.67 ×109/L	129(47%)	45(35%)	84(65%)	
<11.67 ×109/L	145(53%)	36(25%)	109(75%)	
NEU% cut-off value				*p=*0.003
>=89.60%	109(40%)	43(39%)	66(61%)	
< 89.60%	166(60%)	38(23%)	128(77%)	
Administration time (sensitive antibacterial drugs)				*p=*0.258
24h	174(71%)	37(25%)	137(75%)	
48h	31(12%)	3(10%)	28(90%)	
>=72h	41(17%)	9(22%)	32(78%)	
Infection Source				*p* < 0.001
Lung infection	86(27%)	38(44%)	48(56%)	
Skin and soft tissue tissue (Debridement treatment)	75(23%)	20(27%)	55(73%)	
Catheter related infections (Remove the catheter)	78(24%)	13(17%)	65(83%)	
Infectious endocarditis	14(4%)	4(29%)	10(71%)	
Osteomyelitis (Debridement treatment)	25(8%)	1(4%)	24(96%)	
Abdominal infection	11(3%)	7(64%)	5(36%)	
S.aureus drug resistance				*p* = 0.023
MRSA	122(38%)	46(38%)	76(62%)	
MSSA	199(62%)	51(26%)	148(74%)	
qSOFA score				*p* < 0.001
>=2 score	55(24%)	29(53%)	26(47%)	
0 and 1 score	176(76%)	43(24%)	133(76%)	
Antimicrobial agents				*p* = 0.382
Vancomycin	97(41%)	24(25%)	73(75%)	
Levofloxacin or moxifloxacin (Sensitive)	47(29%)	7(15%)	40(85%)	
Cephalosp or carbapenem (Sensitive)	91(20%)	19(21%)	72(79%)	
Hematologic disease or malignant tumor				*p* = 0.067
Yes	48(15%)	20(42%)	28(58%)	
No	273(85)	77(28%)	196(72%)	
Surgical operation of debridement				p=0.06
Yes	33(41%)	3(9%)	30(91%)	
No	48(59%)	12(25%)	36(75%)	
Complicated SAB VS Uncomplicated SAB				p=0.986
Complicated SAB	73(23%)	22(30%)	51(70%)	
Uncomplicated SAB	248(77%)	77(31)	171(69)	

Data are median (IQR) or n (%). p values were calculated by Mann-Whitney U test, χ2 test, or Fisher’s exact test, as appropriate. The classification of complicated SAB 73 cases (23%) included: positive follow−up blood cultures after 48 hours (38 cases), endocarditis (14 cases), and the risk and diagnosis of metastatic foci (21 cases).

## 4 Discussion

*S. aureus* is a leading cause of life-threatening bloodstream infections (BSIs), including sepsis and endocarditis (15). In our 13-year retrospective study of 3,516 BSI cases, *S. aureus* accounted for 9.12% (321/3,516), ranking as the third most common pathogen after E. coli and K. pneumoniae (16). Among the 321 enrolled *S. aureus*-BSI patients (mean age 51.5 ± 21.5 years; range: 3-85), a significant male predominance was observed (male-to-female ratio: 2.7:1). This aligns with the known higher risk associated with occupational exposures in fields such as construction, agriculture, and the military, where skin injury, contact with contaminants, and crowded living conditions are common. The analysis was based on 728 positive blood culture bottles (392 aerobic, 324 anaerobic, and 12 pediatric), with a median time to culture positivity of 14 hours (IQR: 10-23), consistent with reported practices where preliminary susceptibilities are available approximately 16–18 hours after positivity (17).

*S. aureus* is a major human pathogen responsible for a broad spectrum of clinical diseases, including infective endocarditis, osteoarticular infections, skin and soft tissue infections, pleuropulmonary infections, and device-related infections (18). Populations at elevated risk include individuals undergoing hemodialysis, people who inject drugs, those living with HIV, and patients with neutrophil dysfunction, iron overload, or diabetes (18). Severe disease is often associated with breach of cutaneous, mucosal, or implanted device barriers and enters normally sterile sites such as the bloodstream (1). In our study, the most common sources of *S. aureus* bloodstream infection were lung infections (26.8%), catheter-related infections (24.3%), skin and soft tissue infections (23.4%), and urinary tract infections (5.9%). Major comorbidities among patients included hemodialysis due to uremia (21.2%), trauma or burns (8.4%), cerebrovascular disease (7.5%), hematological diseases (7.5%), and cancer or malignant tumors (7.2%). A clear understanding of these primary infection sites and underlying conditions is essential for guiding diagnosis and optimizing treatment strategies.

In our study evaluating PCT, WBC, and NEU% for the diagnosis of *S. aureus* bloodstream infection (BSI), these biomarkers demonstrated excellent discriminative ability to distinguish SAB patients from non−infected controls, with AUC-ROC values of 0.957, 1.000, and 0.913, respectively. The optimal cutoff values were determined as 1.84 ng/mL for PCT, 11.67 × 10^9^/L for WBC, and 89.60% for NEU%. In contrast, a qSOFA score ≥2 showed low sensitivity (23.8%) in predicting *S. aureus* BSI. To our knowledge, no previous literature has reported these findings.

This study characterized 321 *S. aureus* isolates from bloodstream infections (BSIs) and revealed a MRSA prevalence of 38%, which is higher than the 27.1% (95% CI: 23.5–31.0) reported in the WHO Global Antibiotic Resistance Surveillance Report 2025 (19). As anticipated, both MRSA and methicillin-susceptible *S. aureus* (MSSA) exhibited high resistance (≥50%) to penicillin, erythromycin, and clindamycin—a pattern widely documented in the literature and this pattern is associated with common resistance mechanisms (20). Reassuringly, no resistance was detected to key anti-MRSA agents such as linezolid, vancomycin, or tigecycline, consistent with large-scale surveillance data that support their continued efficacy, though ongoing monitoring remains essential (21). Furthermore, a chi-squared test confirmed that the overall resistance rate was significantly higher in the MRSA group than in the MSSA group (α = 0.05/10 = 0.005), and the difference was statistically significant. This broader resistance profile in MRSA is associated with carriage of resistance genes (e.g., mecA) and with efflux pump activity, which correlate with reduced intracellular antibiotic concentrations and multidrug resistance (22).

Among 321 patients with *S. aureus* BSIs, the overall in-hospital mortality rate was 30.2% (97/321). Consistent with previous reports (23, 24), mortality was significantly higher in the MRSA group (38%) than in the MSSA group (26%) (χ² = 3.309, p = 0.048).A notable finding was the acuity of these infections, with 36.1% of deaths occurring within 24 hours, highlighting the critical window for early clinical intervention (25). Survival analysis further corroborated the severity of MRSA infections, showing a significantly lower 10-day survival rate compared to MSSA (0.609 ± 0.054 vs. 0.759 ± 0.039; log-rank p = 0.029). However, among patients who received active antibiotic therapy, further analysis of 10-day survival showed no significant differences between those treated with vancomycin, levofloxacin/moxifloxacin, or cephalosporins/carbapenems (log-rank p = 0.406). This suggests that, within the context of effective treatment, the choice among these specific regimens may yield similar early survival outcomes, an observation that aligns with some real-world data (26).

In our cohort of 321 patients with *S. aureus* BSIs, a substantial proportion (58.0%) were healthcare-associated infections (HAIs), with nearly half (45.7%) of these caused by MRSA. The strong positive correlation between HAI status and MRSA prevalence these associations were no longer statistically significant after applying the χ² test with Bonferroni correction, this finding contrasts with the established view of MRSA as a predominant nosocomial pathogen (27, 28). Furthermore, the correlation between MRSA prevalence and mortality these associations were no longer statistically significant after applying the χ² test with Bonferroni correction, unlike the literature, the role of this factor as an independent risk factor was foreshadowed (29). Our analysis also identified a set of robust, independent predictors of in-hospital mortality. These included markers of critical illness and host vulnerability, such as a qSOFA score ≥2, age ≥60 years, and a pulmonary source of infection, all of which are well-documented in sepsis outcome literature (30, 31). Additionally, biomarkers indicative of a severe systemic inflammatory response—specifically, a procalcitonin level ≥1.84 ng/mL and a neutrophil percentage ≥89.60%—were significant predictors, underscoring their prognostic utility (32). Most critically, the powerful association between ineffective or absent antibiotic therapy and mortality (OR 2.359) underscores a modifiable key factor, a principle strongly emphasized in current international sepsis management guidelines and expert consensus (33).

Because this is an observational study, all reported relationships should be interpreted as associations rather than causal. Correlation results have been described in associative terms throughout, and causal language has been removed.

## Data Availability

The data supporting this study’s findings are available subject to restrictions. As licensed data from inpatients at the 940th Hospital, they originate from institutional HIS and LIS systems. Due to substantial data volume and Chinese-language source records, organized datasets in English translation will be provided upon reasonable request to the corresponding author, with permission from relevant third parties.
